# The influence of deliberate rumination on the post-traumatic growth of college students during the COVID-19 pandemic and the moderating role of self-efficacy

**DOI:** 10.3389/fpubh.2023.1043402

**Published:** 2023-02-02

**Authors:** Yanhua Xu, Guang Yang, Luan Liu, Xinyi Wu

**Affiliations:** ^1^School of Geography and Environment, Jiangxi Normal University, Nanchang, China; ^2^College of Teacher Education, Capital Normal University, Beijing, China

**Keywords:** deliberate rumination, post-traumatic growth, self-efficacy, moderating role, COVID-19

## Abstract

**Objective:**

To understand the relationship between deliberate rumination and post-traumatic growth and the mechanisms affecting this relationship, we constructed an adjustment model to test the impact of deliberate rumination on the post-traumatic growth of college students and the moderating role of self-efficacy during the 2019 COVID-19 pandemic.

**Study design and setting:**

A total of 881 college students from a university of science and technology in Guangdong Province, China, completed a questionnaire that measured deliberate rumination, post-traumatic growth, and self-efficacy. SPSS (version 26) and the PROCESS plug-in (version 4.0) were used for correlation and moderation analyses.

**Results:**

The correlation analysis showed that deliberate rumination was positively correlated with post-traumatic growth (*r* = 0.353, *P* < 0.01) and self-efficacy (*r* = 0.261, *P* < 0.01). Self-efficacy was also positively correlated with post-traumatic growth (*r* = 0.466, *P* < 0.01). In addition, we found that self-efficacy had a regulatory effect on the relationship between deliberate rumination and post-traumatic growth (*R*^2^ = 0.287, *P* < 0.001) and that this effect was significant.

**Conclusion:**

The results show that deliberate rumination can be a positive predictor of post-traumatic growth and can play a certain role in fostering such growth. In addition, self-efficacy is a moderator that plays a buffer role between deliberate rumination and post-traumatic growth. These results contribute to a more comprehensive understanding of the mechanisms that affect post-traumatic growth.

## 1. Introduction

The novel coronavirus epidemic (COVID-19), which began in 2019, has been an unprecedented large-scale traumatic event involving the whole world, and it has brought great trauma to human society (1). The World Health Organization designated it as a public health emergency of international concern (2). In the face of this major emergency, China immediately took measures to encourage people to wear masks when going out and conducted isolation of people in risk areas so as to reduce the risk of virus transmission (3). These prevention measures brought some negative effects on college students such as increased life pressure, causing life regret, and career and life planning (4). Therefore, the academic community became extremely concerned about the negative impact of COVID-19.

One cross-sectional study showed that stress faced by adolescents may lead to physical and mental health challenges and that COVID-19 may lead to the deterioration of adolescents' mental health (5). During the COVID-19 pandemic, the incidence of mental health symptoms such as depression, anxiety, and insomnia was higher than that before the outbreak (6). Therefore, people's mental health has become an important topic of discussion. Rumination, a psychological factor that can be separated into deliberate rumination and intrusive rumination (7), refers to an individual's psychological change through continuous reflection on traumatic events. In general, positive psychological changes tend to be caused by deliberate rumination (8). In this article, we mainly investigate deliberate rumination. Post-traumatic growth, another psychological factor that we examine in this study, refers to improvement in an individual's psychology after experiencing a traumatic event (9).

Deliberate rumination and post-traumatic growth have attracted extensive attention in the research community. Deliberate rumination refers to a person's self-reflection on traumatic events to facilitate the understanding of such events and their impact (10). People who deliberately ruminate have been more likely to achieve higher levels of post-traumatic growth during the COVID-19 pandemic (11), which means that researchers should explore the relationship between the two more deeply.

Although the incidence of mental health symptoms such as depression, anxiety, and insomnia has been higher during the COVID-19 pandemic, people also experienced post-traumatic growth. The existing literature has provided many studies related to deliberate rumination (11, 12). However, few previous studies have investigated the relationship between self-efficacy, deliberate rumination, and post-traumatic growth during the COVID-19 pandemic (8). In order to fully understand post-traumatic growth in academia, we explored the relationship between deliberate rumination and post-traumatic growth, and the regulatory role of self-efficacy in this relationship.

### 1.1. Deliberate rumination as the predictor of post-traumatic growth

Deliberate rumination refers to a conscious thinking process through which people try to understand the cause and significance of events (7). It is commonly believed that deliberate rumination is a positive reflection process (12). It has been demonstrated that deliberate rumination is an active cognitive process including the reshaping of existing schemas to facilitate post-traumatic growth and the positive effect on them after experiencing a traumatic event (13). A term closely related to deliberate rumination is intrusive rumination, and these phenomena are collectively referred to as rumination. From previous studies, we have learned that intrusive rumination can have a negative effect, while deliberate rumination can lead to more positive effects (14). Therefore, we can further study the influence of college students on deliberate rumination.

Korean scholars studied the effects of invasive rumination and deliberate rumination on depression (15). Studies have shown that a high level of intrusive rumination can lead to severe depression, while deliberate rumination triggered by a low degree of intrusive rumination can lead to less severe depression (16). Another study has investigated whether intrusive rumination and deliberate rumination mediate the association between pandemic stress and the severity of depression and anxiety, concluding that intrusive rumination has a mediating effect on the relationship, while deliberate rumination does not (17). Deliberate rumination and intrusive rumination immediately after a traumatic event have been positively correlated with finding meaning (12). Moreover, other studies have shown that depression is negatively correlated with deliberate rumination (18). A study of Chinese patients over 50 years old with esophageal cancer explored the relationship between social support and mental wellbeing, finding that they were negatively correlated with intrusive rumination and positively correlated with deliberate rumination (19). In addition, a study suggests that continuous deliberate rumination is a protective factor against post-traumatic stress disorder and a predictor of post-traumatic growth (20).

Post-traumatic growth is considered to be a form of psychological growth or transformation in individuals after they experience potentially traumatic events (9). A study of post-traumatic growth suggests that the possibility of growth is caused by a high-stress event that seriously challenges or destroys an individual's ideal world (7). A study has shown that in order to stimulate post-traumatic growth, the events experienced must be serious enough to overwhelm the adaptive resources of the individual (21). In creating the term “post-traumatic growth” (9), Tedeschi and Calhoun believed that it is possible to assess whether a person's traumatic and painful experience has shaken their basic life values and world outlook. These positive outcomes include positive perceptions, positive emotions, improved relationships with others, and greater enjoyment of life. Traumatic events may bring great pressure on individuals and lead to post-traumatic stress disorder; however, some people remain resilient, and after experiencing these traumatic events, they may even show personal growth (22).

Post-traumatic stress disorder and post-traumatic growth are contrasting phenomena. The former has a negative effect on individuals, while the latter has a positive effect (23). A Chinese study has shown that individuals may experience post-traumatic stress disorder and post-traumatic growth after a traumatic event (24). The phenomenon of post-traumatic growth is complex and not uniform. Perceived post-traumatic growth has been related to the impairment of mental health (25). Reduced suicidal ideation has been significantly and positively correlated with post-traumatic growth (26), which provides some support for its effectiveness in reducing suicidal ideation (26). The results of a study of the post-traumatic growth of Wenchuan earthquake survivors showed that extroverts tended to obtain more social support in achieving post-traumatic growth (27). A study of the relationship between the self-efficacy and post-traumatic growth of college students during the COVID-19 pandemic showed that those with stronger self-efficacy could experience post-traumatic growth (7). In addition, individuals who challenge core beliefs are more likely to have positive deliberate reflection during sports, and such deliberate rumination can predict the degree of post-traumatic growth (28).

Most previous research has focused on the relationship between rumination (both deliberate and intrusive) and post-traumatic growth. The results have shown that deliberate rumination is more conducive to post-traumatic growth (29). Therefore, we are inclined to encourage deliberate rumination after the experience of traumatic events, so as to enhance our understanding of such events and gain useful experience. However, continuous intrusive rumination can expose people to traumatic cues, thereby encouraging further cognitive processing of a traumatic event, leading to post-traumatic growth (30). In addition, intrusive rumination can have a negative impact on emotional cognitive clarity and a static impact on deliberate rumination. Deliberate rumination can enhance emotional cognitive clarity, thus promoting post-traumatic growth. After experiencing post-traumatic events such as the Wenchuan earthquake, invasive reflection by adolescent survivors increases negative thoughts about the causes and consequences of these events and reduces their ability to separate attention from negative emotional information (20). However, there are few studies on whether deliberate rumination can predict post-traumatic growth (31). Therefore, we propose the following hypothesis:

H1: There is a positive correlation between deliberate rumination and post-traumatic growth for participating college students.

### 1.2. Moderating role of self-efficacy

The concept of self-efficacy was first proposed by Bandura, who proposed the social learning theory (32). It refers to an individual's subjective judgment of his ability to successfully perform a certain behavior (32). Bandura defined two aspects of self-efficacy: the belief that one can succeed in a specific situation or cope with difficulties and challenges in life, and the belief that one can control the extent of one's ability and not affect the lives of others (33). As a social cognitive structure, self-efficacy refers to people's confidence in their ability to control their own functions, overcome difficulties, and perform specific tasks. It determines how people feel, think, motivate themselves, and behave (32). In addition, studies show that self-efficacy can be developed through mastery of experience, social modeling, and persuasion (34, 35).

Numerous studies have shown that many factors interact with self-efficacy. One study showed that working during the COVID-19 pandemic was challenging and stressful for nurses, and that improve their self-efficacy and self-confidence, increase their interaction with patients, and promote the recovery of patients with COVID-19 (1). Institutions and social environments as more supportive workplaces, thus increasing the ability of high self-efficacy people to overcome obstacles, gain a sense of security, or reflect social norms (36). Appropriate social support can improve people's self-efficacy, encourage them to take positive coping measures in the face of difficulties, and thus effectively prevent or reduce the occurrence of psychological stress (31). Physical activity, a complex behavior affected by various personal, social, and environmental factors, may be affected by personal self-efficacy. Social support and exercise self-efficacy are of great significance to the promotion of physical activity among adolescents, a result that can help to formulate effective interventions in this population (37). Self-efficacy can also have a significant impact on perceived social support and resilience (38). Therefore, comprehensive interventions that target perceived social support and self-efficacy can help to improve individual resilience.

By ruminating deliberately on traumatic events, individuals can return to a positive emotional state and further enhance their sense of self-efficacy. Recalling positive events through rumination can increase self-confidence and thus enhance self-efficacy (32). At the same time, individuals can also gain improvement from certain abilities through deliberate rumination. When individuals face traumatic events, those with a high sense of self-efficacy can help themselves to actively recognize and reflect on problems more easily, i.e., engage in rumination (39). Since, based on previous studies, deliberate rumination is the positive aspect of rumination, we infer that deliberate rumination is likely to be positively correlated with self-efficacy.

Relevant studies have shown that self-efficacy has a positive impact on post-traumatic growth (40). After college students experience trauma, their self-efficacy can maintain their self-confidence and awaken their determination and courage to overcome difficulties. Therefore, they can be inspired to maintain an optimistic and upward attitude, take courage from trauma, and achieve post-traumatic growth (41). Children with high self-efficacy can obtain more social support and achieve a higher level of post-traumatic growth, which can reduce the occurrence of behavioral problems (31). Individuals with high self-efficacy tend to rationally handle difficulties and setbacks, keep positive, and meet challenges optimistically.

Many studies have explored the mediating role of self-efficacy. Self-efficacy can mediate and reconcile the relationship between creativity and achievement goals (36). Persons with strong motivation and practical ability can achieve team creativity through collective efficiency (38). During the COVID-19 pandemic, college students with high self-efficacy have obtained more positive psychological changes in the growth process after trauma, thus enhancing creativity (7). In addition, when individuals engage in deliberate rumination, they tend to focus on the positive significance of traumatic events and establish more ambitious goals and beliefs. During this process, they are likely to enhance their self-efficacy and thus achieve post-traumatic growth (42). Based on the existing literature and the above three hypotheses, we infer that self-efficacy may play a regulatory and buffering role in the relationship between deliberate rumination and post-traumatic growth. Therefore, we propose self-efficacy as a potential regulator.

H2: Self-efficacy plays a regulatory role in the relationship between deliberate rumination and post-traumatic growth.

**Figure 1:**
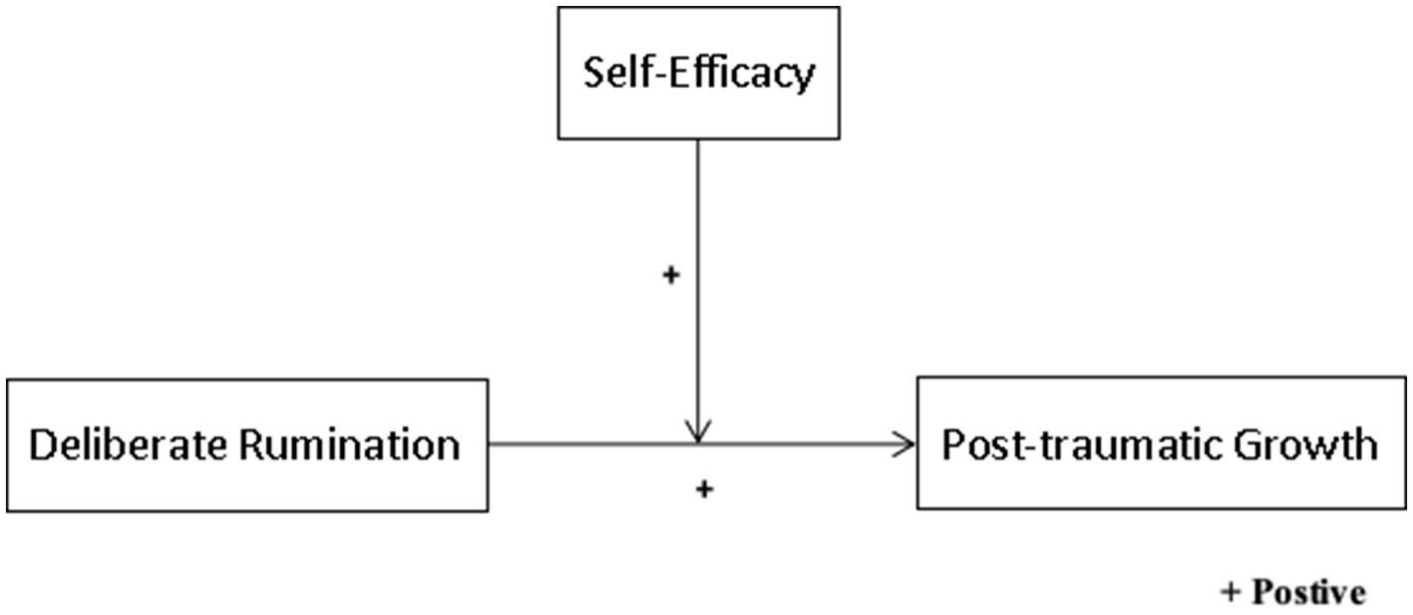
Graphic representation of relationships between study variables.

Figure 1 presents the regulatory model proposed in the four hypotheses and depicts the relationship between independent, dependent, and regulatory variables.

## 2. Materials and methods

### 2.1. Participants and procedures

The participants in this study were enrolled in a technical college in Guangdong Province, China, that serves more than 20,000 regular full-time university students. Of these, 918 university students filled in the questionnaire. After data collection, we deleted 37 participants who were not from Guangdong, so the actual number of questionnaires analyzed was 881. Among these, 317 were male (35.1%) and 564 were female (64.0%). Prior to the questionnaire, exploratory focus group interviews were conducted with a selection of students from the college. They were experiencing a severe outbreak of New Coronary Pneumonia in Guangdong Province at the time, and therefore selecting from among them for the exploratory focus group interviews may have added value to our findings. This was because almost all but one region in Guangdong Province was experiencing cases at the time. Due to the severe form of the epidemic, university students were attending classes online at the time. Therefore, we ended up recruiting 20 students and divided them into four focus groups of 4–6 students each. They came from English, Human Resource Management, Financial Management, Computer Science, and Chinese Language and Literature, all of whom had different logical thinking and needed to be properly guided by the facilitators to speak their minds. As this was a particular time in the New Coronary Pneumonia epidemic, online interviews were conducted using a web conference format. The facilitator develops questions in advance to guide the focus group through a full and detailed discussion of relevant topics, draws on their understanding and perspectives on the subject, clarifies their emotional profile and mental state, and provides a basis for the development of the questionnaire. To ensure the depth and validity of this interview discussion, participants will be asked to turn on the microphone and camera throughout. With the consent of the participants, the entire interview will be recorded and the facilitator will be responsible for collating the interview and outputting it into written notes. In the process of collating the written notes, the transcribed text of the interviews was sorted and simplified, with keywords extracted from the text and divided into different modules. The keywords were then coded and divided into further sub-modules and the questionnaire was designed to obtain further detailed data based on what was contained in these refined sub-modules. Furthermore, based on the results of the interviews, we may be surprised to find that 62% of the respondents indicated that they would feel depressed during the COVID-19 outbreak. They said that they would unconsciously think about events related to the epidemic and have difficulty concentrating; they would be distracted from doing things well when they hear about the epidemic; they would also feel regret and sadness for the impact the epidemic has brought to their lives and studies. After experiencing the Newcastle Pneumonia epidemic, most of the university students said that they would cherish life more and enjoy life more in the future; they also felt the care and concern from their relatives, friends, teachers, and medical staff, and felt that the world is full of love. When asked if they thought humans could overcome the epidemic, they all expressed their affirmation and said they could contribute their share to the fight against the epidemic.

This study used a related design with a web-based questionnaire as a data collection method. The questionnaire was completed between 10 April and 15 June 2020. During the break of public classes in the same grade, researchers showed university students the two-dimensional code to fill in the questionnaire through multimedia devices. University students who volunteered needed only to scan the QR code, enter the questionnaire interface, respond to the items, and click submit. (A QR code is a bar code readable by the cameras of mobile phones, tablets, and other devices. In China, QR codes are widely used to open specific link interfaces and carriers for various applications.) It should be emphasized that before inviting university students to scan the code, we explained the purpose of the research in detail. All university students gave their consent to participate in this research study and completed a written consent form, which was later collected with the questionnaire.

### 2.2. Materials

The questionnaire used in this study consisted of 51 items, divided into four sections: demographic information and scales that measured post-traumatic growth, self-efficacy, and deliberate rumination. The demographic information included gender, home address, and major. The three scales, originally developed in English, were translated into Chinese for this study. In order to improve the quality of translation, we adopted the back-translation method (43); i.e., the first research man translated the English-language scale into Chinese, then a second researcher translated the Chinese-language version back into English, and finally a third researcher compared the original version, the translated version, and the back-translated version, and evaluated the accuracy of the Chinese-language version. Before finalizing the questionnaire, the language of the Chinese-language version was revised and optimized to ensure the effectiveness of the scale.

#### 2.2.1. Deliberate-rumination scale

This study used the deliberate rumination scale in the Event-related Rumination Inventory, developed by Cann et al. (44). This scale comprises 10 items. After discussion and modification, we decided on 20 statements (e.g., “I don't want to, but I still think of this thing” and “I often think about it, and it's hard to stop”). Participants rated each statement on one of four levels (1 = *Not at all*, 4 = *Always*). For this study, the internal consistency coefficient of the scale was 0.913.

#### 2.2.2. Post-traumatic growth scale

This study used the Chinese version of the Posttraumatic Growth Inventory translated by Geng et al. (45) for the Chinese context. The scale includes 21 items that measure interpersonal relationships (e.g., “I know that when I have difficulties, I can rely more on others” and “I feel I am closer to others”), new possibilities (e.g., “I develop new hobbies” and “I have established a new direction for my own life path”), personal strength (e.g., “I know I can handle problems better now” and “I can make my life better”), spiritual change (e.g., “I have more confidence” and “I have more understanding of spiritual things”), and appreciation of life (e.g., “I can treasure every day more” and “I can treasure the value of life more”). Participants rate the degree of feeling, reaction, or identification on a 6-point scale (1 = *No change*, 6 = *Greatly changed*). For this study, the internal consistency coefficient of the scale was 0.958.

#### 2.2.3. Self-efficacy scale

The self-efficacy scale developed by Schwarzer et al. (46) was used in this study. There are 10 items in the original scale, which has been shown to have good reliability and validity in a sample of Chinese university students. To better fit the specific situation of participating university students and the needs of this study, we decided to remove the last three items after group discussion. Therefore, the number of items was reduced to seven (e.g., “If I try my best, I can always solve problems” and “I am confident that I can effectively deal with any unexpected things”). Participants rated each statement at one of four levels (1 = *Completely incorrect*, 4 = *Completely correct*). For this study, the internal consistency coefficient of the scale was 0.875.

### 2.3. Data analysis

We used SPSS 26.0 for data processing and analysis. Since data collection was done by self-report, and in order to ensure the validity of the data, we applied the Harman single-factor test before data processing to determine if common method bias was present, in order to ensure the validity of the data (47). The results of testing the questionnaire items related to the three variables showed that the eigenvalues of four factors were >1. The contribution rate of the factors to the total variance was 63.682%, and the variation interpretation rate of the first factor was only 39.874%, which did not meet the critical standard of 40% (48). In other words, there was no significant common methodological bias in this study.

We then conducted descriptive analysis, correlation analysis, and model testing of the data based on the research hypotheses. First, we examined the concentration and dispersion of data using descriptive analysis. Then, we analyzed correlations among the variables and tested the relationship among independent, dependent, and regulatory variables by calculating Pearson correlation coefficients. We further investigated the research hypotheses using the PROCESS (version 4.0) plug-in of SPSS to test the moderating effect of the model. (The PROCESS plug-in was developed by Hayes (49) for moderation and moderation analysis based on path analysis and their combination).

## 3. Results

### 3.1. Descriptive statistics and correlation analysis

SPSS 26.0 was used to calculate and test means, standard deviations (Table 1), and correlations (Table 2) of study variables. Deliberate rumination was significantly and positively correlated with self-efficacy (*r* = 0.216, *P* < 0.01) and post-traumatic growth (*r* = 0.353, *P* < 0.01). In addition, post-traumatic growth was positively correlated with self-efficacy (*r* = 0.466, *P* < 0.01). At a moderate level, the correlation between post-traumatic growth and self-efficacy was the highest. It can be seen that the subsequent moderating effect test is supported by correlation analysis.

**Table 1:** Mean scores and standard deviations for variables, according to gender.

**Variable**	***N***	***M***	**SD**
Deliberate rumination	881	2.012	0.018
Male	317	2.044	0.030
Female	564	1.995	0.022
Post-traumatic growth	881	3.301	0.034
Male	317	3.392	0.057
Female	564	3.250	0.042
Self-efficacy	881	2.318	0.018
Male	317	2.307	0.031
Female	564	2.324	0.022

**Table 2:** Correlations among variables.

**S. No**.	**Variables**	**Deliberate rumination**	**Post-traumatic growth**	**Self-efficacy**
1.	Deliberate rumination	–		
2.	Post-traumatic growth	0.353^**^	–	
3.	Self-efficacy	0.216^**^	0.466^**^	–

N = 881,

** p < 0.01.

### 3.2. Moderating effect

In order to test H4, we examined whether self-efficacy had a moderating role in the relationship between deliberate rumination and post-traumatic growth. SPSS PROCESS (version 3.2) model 1 was used to perform adjustment analysis, with deliberate rumination as the independent variable, post-traumatic growth as the dependent variable, and self-efficacy as the moderating variable. The results (Table 3) showed that self-efficacy had a significant effect on post-traumatic growth (β = 0.761, *P* < 0.001), and 95% CI (0.654, 0.868) excluding 0. The interaction between deliberate rumination and self-efficacy had a significant effect on self-efficacy (β = −0.183, *P* < 0.05), 95% CI (−0.356, −0.011), which indicates that the direct effect of deliberate rumination and post-traumatic growth is moderated by self-efficacy.

**Table 3:** Moderated mediating effect of general self-efficacy on post-traumatic growth.

**Predictor**	**Post-traumatic growth**					
	β	**SE**	***t***	***p***	**95% CI (lower)**	**95% CI (upper)**
DR	0.541	0.058	9.281	0.000	0.427	0.656
SE	0.761	0.055	13.958	0.000	0.654	0.868
DR*SE	−0.183	0.088	−2.082	0.038	−0.356	−0.011
*R* ^2^	0.287					
*F*	117.913^***^					

Analyses conducted using PROCESS model 1. N = 881.

*** p < 0.001.

DR, deliberate rumination; SE, self-efficacy.

**Figure 2:**
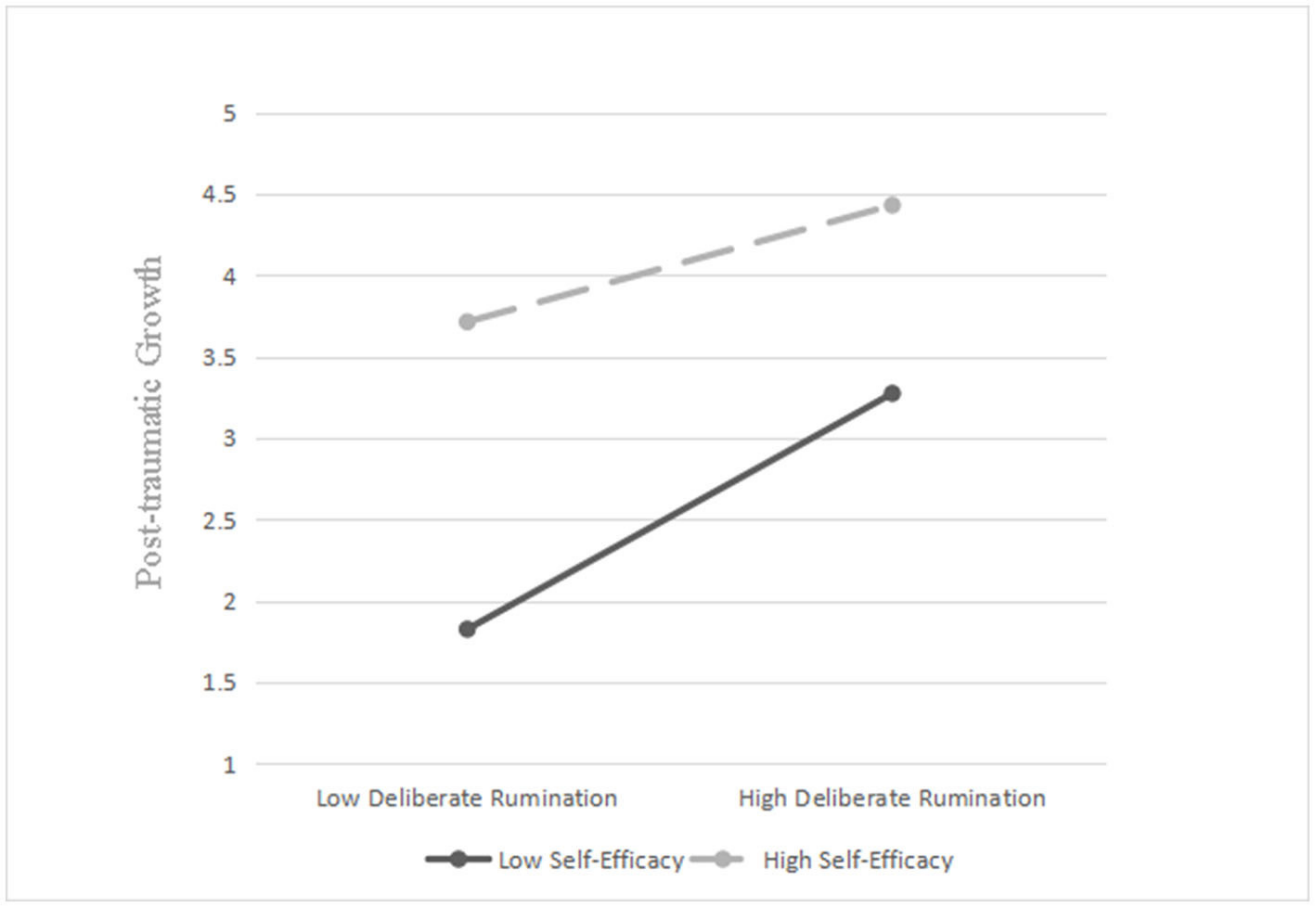
Relationship between deliberate rumination and post-traumatic growth for high and low levels of self-efficacy.

In order to further analyze the moderating effect of self-efficacy, we divided self-efficacy scores into a low group (M – 1 SD) and a high group (M + 1 SD) and conducted a simple slope analysis. The results (Table 4) show that 95% confidence intervals do not include 0, and that self-efficacy affects the strength of the relationship between deliberate rumination and post-traumatic growth. Figure 2 maps the scores of the two groups (low-level and high-level self-efficacy). When self-efficacy is higher (high group), the direct effect of deliberate rumination on post-traumatic growth is stronger; when self-efficacy is low (low group), the direct effect of deliberate rumination on post-traumatic growth is weaker.

**Table 4:** Conditional effect at specific levels of deliberate rumination.

**Conditional effect of self-efficacy**	**Estimate**	**SE**	***t***	***p***	**95% CI**	
					**Lower**	**Upper**
−1 SD	0.640	0.084	7.614	<0.001	0.475	0.805
M	0.541	0.058	9.281	<0.001	0.427	0.656
+1 SD	0.443	0.065	6.828	<0.001	0.316	0.570

## 4. Discussion

### 4.1. Discussion of results

In this study, we established a regulatory model to explore the relationship between college students' deliberate rumination and post-traumatic growth during the COVID-19 pandemic, and the role of self-efficacy as a potential regulator of this relationship. It was found that, for participants, deliberate rumination was positively correlated with post-traumatic growth; deliberate rumination was positively correlated with self-efficacy; self-efficacy was positively correlated with post-traumatic growth; and self-efficacy played a regulatory role between deliberate rumination and post-traumatic growth. These results validate the research hypothesis, explain the moderation model, and are consistent with previous research findings.

#### 4.1.1. The positive association between deliberate rumination and post-traumatic growth

The results are consistent with H1 and the existing literature, indicating that deliberate rumination is positively correlated with post-traumatic growth. In other words, the more deliberate that rumination is, the more post-traumatic growth that can be achieved. It has been documented that individuals can achieve post-traumatic growth through deliberate rumination (20), and studies have confirmed that the higher the level of deliberate rumination, the more obvious the effect of post-traumatic growth (50). This means that the more university students ruminate deliberately during the COVID-19 pandemic, the more they will experience post-traumatic growth. Hyu Jung Huh et al. demonstrated that deliberate rumination may be positively correlated with post-traumatic growth and that during deliberate rumination, people are better able to understand traumatic events and effects and achieve post-traumatic growth (10). Mostarac and Brajković (51) also demonstrated that deliberate rumination can better facilitate post-traumatic growth. In addition, it is also worth mentioning that deliberate rumination has a high level of correlation with post-traumatic growth, which is only predicted by deliberate rumination (52). Therefore, if university students have more opportunities to engage in deliberate rumination about their regrets and to overcome their tragic experiences during traumatic events like the COVID-19 pandemic, they are more likely to achieve post-traumatic growth.

In summary, the results of this study show that active rumination predicts post-traumatic growth, suggesting that the more active rumination behaviors college students experience when exposed to a traumatic event such as the COVID-19 pandemic, the more likely they are to also experience post-traumatic growth.

#### 4.1.2. The moderating role of self-efficacy

First, the results of the study show that deliberate rumination has a positive predictive effect on self-efficacy (8). This means that the stronger the inclination to ruminate, the higher the level of self-efficacy. Previous studies have shown a positive relationship between self-efficacy and deliberate rumination (53, 54). Gilliam (55) conducted a study on depressed patients and showed that rumination has an effect on self-efficacy in controlling disturbing thoughts and is consistent with the social cognitive theory proposed by Bandura (32). Zeng et al. (3) studied the relationship between rumination and self-efficacy in college students after the New Crown outbreak and also suggested that deliberate rumination has a positive relationship with self-efficacy. Although this result has been less discussed in most previous studies, there are previous findings that indicate that deliberate rumination is positively related to self-efficacy, with more deliberate rumination being associated with greater self-efficacy (56, 57), and Sanchez-Teruel and Robles-Bello (53) have also shown that deliberate rumination positively predicts self-efficacy. This result shows that the more university students engage in deliberate rumination during the COVID-19 pandemic, the more they can feel the determination to overcome this traumatic event, repeatedly experience the moment of courage and the spirit of struggle, and improve their self-confidence and thus their self-efficacy.

Second, the results confirmed that self-efficacy can positively predict post-traumatic growth. These results are consistent with those of previous studies. Relevant studies have shown that self-efficacy is significantly and positively correlated with post-traumatic growth (58). This means that persons with a strong sense of self-efficacy are more likely to achieve post-traumatic growth. Such people also tend to be optimistic in the face of challenges, brave when responding to them, and always full of confidence and positive energy, which helps to achieve more positive post-traumatic growth (33). Therefore, individuals with a high level of self-efficacy have the confidence to achieve a sense of control over negative emotions. They are more able to develop a positive attitude and enhance their ability to cope with stress and a disturbing living environment, thus promoting post-traumatic growth. To sum up, in the context of the COVID-19 pandemic, the higher the self-efficacy of college students, the less they are vulnerable to its trauma. The more able they are to face this trauma, the better they can control their lives and stay positive. Thus, university students with a sense of self-efficacy can achieve post-traumatic growth faster and experience the pandemic with less difficulty.

To sum up, these results confirmed H2, revealing that self-efficacy plays a regulatory role in the relationship between deliberate rumination and post-traumatic growth. This result means that, during the COVID-19 pandemic, self-efficacy can help college students improve their ability to achieve post-traumatic growth during deliberate rumination. The results show that university students with a high level of self-efficacy are less likely to be negatively affected by deliberate rumination, and university students with low self-efficacy are more likely to be negatively affected. Many previous studies have shown that self-efficacy is an important protective factor, that can reduce the negative impact of deliberate rumination (40). Among them, one study showed that university students with a high level of self-efficacy are more inclined to be positively influenced by deliberate rumination, thus helping them achieve post-traumatic growth. In addition, many related studies have also confirmed that university students can strive to play a positive role in deliberate rumination by virtue of self-efficacy beliefs (59, 60). Other studies have shown that, when facing difficult life challenges and major life crises, maintaining a positive attitude is conducive to coping with and overcoming difficulties with optimism, thereby achieving post-traumatic growth (41, 61). More specifically, after experiencing the trauma of the COVID-19 pandemic, the self-efficacy of an individual student can regulate the relationship between their deliberate rumination about the experience and their post-traumatic growth. In the process of deliberately ruminating about such an experience, the student with stronger self-efficacy can better reflect on their determination and courage to overcome difficulties, enhance their self-confidence, maintain their optimism and progress, and achieve post-traumatic growth. Therefore, self-efficacy regulates and enhances the relationship between deliberate rumination and post-traumatic growth.

### 4.2. Implications

This study enriches the understanding of deliberate rumination and post-traumatic growth among Chinese college students during the COVID-19 epidemic, and the results are significant from both a theoretical and a practical perspective.

Theoretically, this study links deliberate rumination to post-traumatic growth, elaborates on previous theories, and deepens understanding of the positive effect of deliberate rumination on the post-traumatic growth of college students. In addition, the results with respect to the moderating role of self-efficacy confirm that, during the COVID-19 pandemic, college students with strong self-efficacy could achieve more post-traumatic growth by engaging in deliberate rumination. This study, therefore, enriches previous theoretical and empirical research on the relationship between deliberate rumination, self-efficacy, and post-traumatic growth, enabling researchers to develop a deeper understanding of their relationship and laying the groundwork for further research.

From a practical perspective, understanding the relationship between the three variables investigated in this study may help researchers to better understand the mechanisms by which post-traumatic growth occurs through deliberate rumination. The findings suggest that university students tend to engage in deliberate rumination after experiencing trauma and that self-efficacy plays a moderating role in the relationship between deliberate rumination and post-traumatic growth. This has important practical implications for our daily lives. If a university student has experienced a traumatic event, his or her close relatives, friends, and teachers should take active guidance measures to help them increase the frequency of deliberate rumination and enhance their sense of self-efficacy in order to facilitate their post-traumatic growth. At the same time, we recommend that universities, teachers, and parents organize appropriate activities to enhance the self-efficacy of university students so that they can face traumatic events more calmly in the future.

### 4.3. Limitations and future directions

This study has certain limitations. First of all, all participants were from one region and one college, which may affect the generalization of the results because the sample may not be representative. A follow-up study could expand the scope of sampling to different universities nationwide. Second, a cross-sectional design was used, and differences over time were not measured. In future research, researchers could design a longitudinal study and examine possible changes in the variables over a long period of time. In addition, researchers could study the relationship between various aspects of deliberate rumination, post-traumatic growth, and self-efficacy, as well as control variables such as professional and economic status, so as to provide more detailed data for follow-up studies.

## 5. Conclusion

We designed a regulatory model to explore the relationship between deliberate rumination and post-traumatic growth, and the role of self-efficacy in regulating this relationship. We found that deliberate rumination was a positive predictor of post-traumatic growth among participating college students, i.e., college students with more deliberate rumination tend to be better able to achieve post-traumatic growth. Those with a higher frequency of deliberate rumination exhibited a stronger self-efficacy than those who engaged in deliberate rumination less often. In addition, those participants with stronger self-efficacy were more likely to achieve post-traumatic growth than those with a lower level. We conclude that self-efficacy positively regulates the relationship between deliberate rumination and post-traumatic growth.

## Data availability statement

The raw data supporting the conclusions of this article will be made available by the authors, without undue reservation.

## Ethics statement

The studies involving human participants were reviewed and approved by the Ethics Committee of Capital Normal University. The patients/participants provided their written informed consent to participate in this study.

## Author contributions

YX and GY designed the research and reviewed and edited the manuscript. LL, XW, YX, and GY reviewed the literature and analyzed the data. LL, XW, YX, and GY wrote the manuscript. All authors contributed to the article and approved the submitted version.
